# miR-1908 Dysregulation in Human Cancers

**DOI:** 10.3389/fonc.2022.857743

**Published:** 2022-04-07

**Authors:** Jinze Shen, Yuchen Wu, Wenjing Ruan, Feng Zhu, Shiwei Duan

**Affiliations:** ^1^ Department of Clinical Medicine, Zhejiang University City College School of Medicine, Hangzhou, China; ^2^ Department of Clinical Medicine, The First School of Medicine, Wenzhou Medical University, Wenzhou, China; ^3^ Sir Run Run Shaw Hospital, College of Medicine, Zhejiang University, Hangzhou, China

**Keywords:** miR-1908, ceRNA, cancer, diagnosis, prognosis

## Abstract

MiR-1908 is a miRNA located in the intron of the fatty acid desaturase 1 (FADS1) gene. The expression level of miR-1908 is abnormal in many diseases such as cancer. miR-1908 can inhibit the expression of at least 27 target genes by binding to the 3’ untranslated region (3’ UTR) of target genes. miR-1908 is involved in the biological processes of cell proliferation, cell differentiation, cell apoptosis, cancer cell invasion, and metastasis. The expression of miR-1908 is regulated by 11 factors, including lncRNA HOTTIP, adipokines (TNF-α, leptin, and resistin), NF-κB, free fatty acid (FFA), cholesterol, stearoyl-CoA desaturase (SCD1), immune-related transcription factors (STAT1, RB1, and IRF1). The expression of miR-1908 is also affected by the anticancer drug OSW-1, growth hormone (GH), and the anticonvulsant drug sodium valproate. In addition, the aberrant expression of miR-1908 is also related to the prognosis of a variety of cancers, including non-small cell lung cancer (NSCLC), ovarian cancer (OC), breast cancer, cervical cancer, glioma, high-grade serous ovarian carcinoma (HGSOC), osteosarcoma, etc. This article summarizes the abnormal expression pattern of miR-1908 in various diseases and its molecular regulation mechanisms. Our work will provide potential hints and direction for future miR-1908-related research.

## Introduction

MicroRNA (miRNA) is a highly conserved non-coding RNA with a length of about 20-25 nucleotides. miRNA can recognize the 3’untranslated region (3’ UTR) of target mRNA and inhibit post-transcriptional gene expression (1). In cancer, miRNA can regulate cell proliferation, growth, differentiation, and apoptosis, and is closely related to the diagnosis and prognosis of cancer (2). In addition, the molecular mechanism of miRNA provides a theoretical basis for the development of related drugs (3).

Hsa-mir-1908 is located in the first intron of the fatty acid desaturase 1 (FADS1) gene (4). In 2008, researchers first reported that miR-1908 is highly expressed in undifferentiated H1 human embryonic stem cells (H1 hESC) (5). miR-1908 is abnormally expressed in various diseases, especially cancer. Studies have shown that miR-1908 can target at least 27 genes and is involved in multiple cancer-related signaling pathways, including regulation of the MAPK signaling pathway (6), TGF-β signaling pathway (1, 7), PI3K/AKT signaling pathway (8, 9), and AMPK signaling pathway (10). miR-1908 regulates cell behaviors such as cell proliferation, differentiation, apoptosis, invasion and metastasis, and exosome secretion by regulating downstream genes, and plays an important role in the occurrence and development of various diseases such as cancer.

The expression of miR-1908 is not closely related to its host gene FADS1 (4) but is regulated by 11 upstream factors including lncRNA HOTTIP, adipokines (TNF-α, leptin, and resistin), NF-κB, free fatty acid (FFA), cholesterol, stearoyl-CoA desaturase (SCD1), immune-related transcription factors (STAT1, RB1, and IRF1). Abnormal expression of miR-1908 is associated with various diseases, as well as the resistance to multiple anticancer drugs.

Although miR-1908 is a relatively newly discovered miRNA, more and more molecular mechanisms of miR-1908 have been discovered recently (11, 12). miR-1908 plays an important role in many human diseases, especially cancer, and there is no systematic description of miR-1908. This article summarizes the abnormal expression of miR-1908 in cancer and other diseases and its molecular regulation mechanism.

## miR-1908 Is Abnormally Expressed in Various Diseases

As shown in **Table 1**, miR-1908 is abnormally expressed in various diseases, which comprise the nervous system, cardiovascular system, immune system, exercise system, respiratory system, digestive system, urinary system, reproductive system, etc.

**Table 1:** Aberrant expression of miR-1908 in cancers and other diseases.

System	Disease	Type of miRNA	Expression of miRNA	Level	Reference
Nervous system	Glioma	miR-1908-5p	upregulated	tissue and cell	(8)
		miR-1908-5p	upregulated	tissue	(13)
		miR-1908-5p	downregulated	tissue	(14)
	AD	miR-1908-5p	upregulated	blood cell	(15)
	BD	miR-1908-5p	downregulated	whole blood	(16)
	UP	miR-1908-5p	no difference	whole blood	
Cardiovascular system	AMI	miR-1908-5p	downregulated	tissue	(1)
	AHS	miR-1908-5p	downregulated	serum	(17)
	IS	miR-1908-5p	downregulated	serum	(18)
Immune system	RA	miR-1908-5p	downregulated	exosome	(19)
Motor system	Osteosarcoma	miR-1908-5p	upregulated	tissue	(12)
		miR-1908-5p	upregulated	tissue and cell	(9)
	Low-energy fracture	miR-1908-5p	upregulated	BM	(20)
Respiratory system	NPC	miR-1908-5p	upregulated	serum and exosome	(21)
	NSCLC	miR-1908-5p	downregulated	cell	(22)
		miR-1908-5p	downregulated	tissue	(23)
Digestive system	HCC	miR-1908-5p	downregulated	plasma	(24)
	HBV infection	miR-1908-5p	upregulated	tissue	(25)
Urinary system	Renal fibrosis	miR-1908-5p	downregulated	tissue	(7)
Reproductive system	Cervical cancer	miR-1908-5p	upregulated	tissue and cell	(26)
	EC	miR-1908-5p	no difference	tissue	(27)
	HGSOC	miR-1908-5p	downregulated	serum	(28)
	PCa	miR-1908-5p	downregulated	cell	(29)
	SSCs	miR-1908-3p	upregulated	cell	(30)
Other	Chordoma	miR-1908-5p	downregulated	tissue	(6)
	LD formation	miR-1908-5p	upregulated	tissue	(31)
	Scar formation post-burn	miR-1908-5p	upregulated	tissue	(32)
	Obesity	miR-1908-5p	downregulated	tissue and cell	(33)
		miR-1908-5p	upregulated	tissue and cell	
		miR-1908-5p	downregulated	cell	(34)
		miR-1908-5p	upregulated	cell	
		miR-1908-5p	upregulated	cell	(4)
	Melanoma	miR-1908-5p	upregulated	cell	(35)
		miR-1908-5p	downregulated	tissue	(36)
	Breast cancer	miR-1908-3p	upregulated	tissue and serum	(37)

AD, Alzheimer’s disease; BD, bipolar disorder; UP, unipolar disorder; BDV, Borna disease virus; AMI, acute myocardial infarction; IS, ischemic stroke; NPC, nasopharyngeal carcinoma; NSCLC, non-small cell lung cancer; HCC, hepatocellular carcinoma; HBV, hepatitis B virus; EC, endometrial cancer; PCa, prostate cancer; SSCs, spermatogonial stem cells; LD, lipid droplet; no difference, including no difference, the difference is not statistically significant; BM, bone marrow.

In peripheral blood, the abnormally high expression of miR-1908 is associated with the risk of Alzheimer’s disease (AD) (15), while the abnormally low expression of miR-1908 is associated with the risk of acute bipolar disorder (BD) (16). In serum, low expression of miR-1908 is associated with acute heart failure (AHF) (17), large artery atherosclerosis (LAA), lacunar infarction (LAC), the stroke of unknown etiology (SUE) (18), and other diseases. The high expression of miR-1908 in serum is related to the risk of NPC (21) and breast cancer (37).

Abnormal expression of miR-1908 also exists in exosomes from multiple sources. In exosomes derived from NPC tumor cells, the expression of miR-1908-5p is elevated (21). The expression level of miR-1908-5p is decreased in the circulating exosomes of patients with hepatocellular carcinoma (HCC) (24). The expression level of miR-1908-5p is down-regulated in synovial fibroblast-derived exosomes of patients with rheumatoid arthritis (RA) (19).

The abnormal expression of miR-1908 in bone marrow tissue is also related to a variety of pathological processes. The high expression of miR-1908 in the bone marrow is associated with the risk of fracture in patients with type 2 diabetes (20). In addition, during the adipogenesis of human mesenchymal stem cells (31), the expression level of miR-1908-5p in bone marrow tissue increases.

miR-1908 also exhibits abnormal expression in a variety of pathological tissues and cells. In pathological tissues or disease cell lines, the abnormally high expression of miR-1908 is associated with glioma (8, 13), osteosarcoma (9, 12), breast cancer (37), prostate cancer (29), and hepatitis (25). However, the abnormally low expression of miR-1908 is also associated with a variety of diseases, including NSCLC (22, 23), cervical cancer (26), high-grade serous ovarian cancer (28), chordoma (6), melanoma (36), acute myocardial infarction (AMI) (1), and renal fibrosis (7).

In addition, the expression of miR-1908 is closely related to the progression of tumors. For example, the high expression of miR-1908 is significantly related to the malignant grade of glioma cells (8, 14) and the metastasis of melanoma cells (35).

Finally, the abnormal expression of miR-1908 is also related to the differentiation of adipocytes. During the differentiation process of human multipotent adipose-derived stem cells (hMADS cells) and human visceral preadipocytes (HPA-V), the expression level of miR-1908-5p decreased in the first 4 days and then continued to increase, until fully differentiated on the 15th Day into the fat cells (33, 34). However, some studies have shown that the expression of miR-1908-5p always increases during the differentiation of HPA-V (4). Besides, the expression level of miR-1908-5p in the subcutaneous adipose tissue of obese people is lower than that of normal people, while the expression level of miR-1908-5p in visceral adipose tissue is higher than that of normal people (33).

miR-1908 is highly expressed in cancer patients such as NPC, osteosarcoma, cervical cancer and breast cancer. The expression of miR-1908 is low in cancer patients such as NSCLC, HCC, HGSOC, PCa and chordoma. The aberrant expression pattern of miR-1908 in gliomas and melanomas remains controversial, possibly due to differences in tissues and cell types as well as differences in the tumor cell microenvironment. In addition, there are many upstream regulators of miR-1908, which also leads to differences in the expression patterns of miR-1908 in various cancers. However, the differences in the expression patterns of miR-1908 in the same type of cancer still require more experimental evidence to determine.

## The Influence of miR-1908 on Cell Behaviors

**Figure 1:**
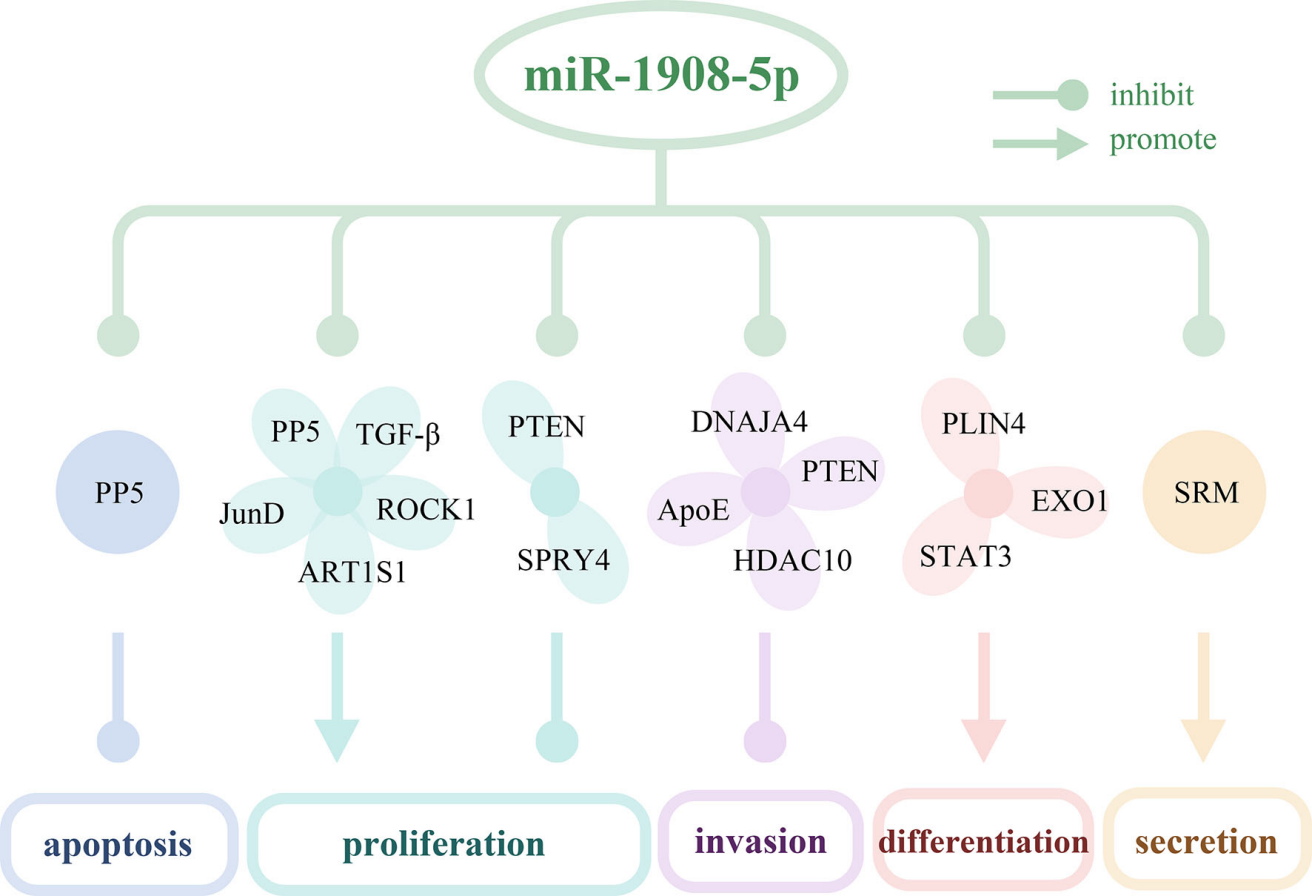
The targeted genes of miR-1908-5p and their effects on cell behaviors. miR-1908-5p regulates cell apoptosis, proliferation, invasion, differentiation, secretion of exosomes by targeting 16 genes.

As shown in **Table 2** and **Figure 1**, miR-1908 can target 23 genes related to cell behaviors. These target genes are involved in the MAPK signaling pathway (6) and the PI3K/AKT signaling pathway (8, 9). By targeting and regulating multiple genes, miR-1908 plays an important role in a variety of cell behaviors such as cell proliferation, cell differentiation, apoptosis, cancer cell invasion and metastasis, and extracellular vesicle secretion.

**Table 2:** Direct target and downstream factors of miR-1908 and the regulation effects *in vivo* and *in vitro* in various diseases.

Type of diseases		Type of miRNA	Direct target	Downstream factors	Effect *in vivo*	Effect *in vitro*	Reference
Cancer	Glioma	miR-1908-5p	PTEN	AKT/FOXO3a pathways, AKT/mTOR pathways	accelerate tumor growth and induced an increase in tumor weight and volume	promote proliferation and invasion of glioma cells (A127, SW1783, U87, U373, LN-229, SW1088, Hs683, HFU251, SNB19, and T98G)	(8)
		miR-1908-5p	—	Bcl-2/Bax	—	enhance activity of MMP-2, promote proliferation and invasion of glioma (U251) cells, and inhibit apoptosis of U251 cells	(13)
			SPRY4	RAF1			
	NSCLC	miR-1908-5p	PP5	—	inhibit growth of tumor	suppress NSCLC cells (SPC-A1) proliferation and induce SPC-A1 cells apoptosis	(23)
		miR-1908-5p	AKT1S1	RP-p53-p21 tumor-suppressing pathway	—	suppress proliferation of NSCLC cells (SK-MES-1, A549, and NCI-H460)	(22)
	Osteosarcoma	miR-1908-5p	PTEN	PI3K/AКТ pathways	increase tumor volume and weight	promote migration, proliferation and self-renewal ability of osteosarcoma cells (143B, U2OS, MG63, and SAOS2)	(9)
		miR-1908-5p	ROCK1	—	—	suppress proliferation of osteosarcoma cells (HOS, MG63, G293, SAOS2, and U2OS)	(38)
	Cervical cancer	miR-1908-5p	HDAC10	—	—	promote growth, invasion, proliferation and colony formation of cervical cancer cells (Ca-Ski, SiHa, and C-4 I)	(26)
	EOC	miR-1908-5p	—	—	—	reduce cell viability of EOC cells (A2780 and SK-OV-3)	(28)
	PCa	miR-1908-5p	SRM	EV	—	promote extracellular vesicle secretion in PCa cells (22Rv1)	(29)
	Chordoma	miR-1908-5p	TGF-β1, JunD	—	—	inhibit chordoma tissue proliferation	(6)
	Melanoma	miR-1908-5p	ApoE, DNAJA4	LRP1,LRP8	promote metastatic invasion, endothelial recruitment, and angiogenesis	enhance capacity of recruit endothelial cells in highly metastatic melanoma cells (MeWo-LM2)	(35)
	Breast cancer	miR-1908-3p	ID4, LTBP4, GPM6B, RGMA, EFCAB1, ALX4, OSR1, PPARA	—	—	promote proliferation, migration, and invasion of breast cancer cells (MCF-7)	(37)
Others	SSCs	miR-1908-3p	KLF2	—	—	promote DNA synthesis, proliferation and inhibit apoptosis of the human SSC line	(30)
	Low-energy fracture	miR-1908-5p	EXO1	—	—	inhibit proliferation and osteogenic differentiation of BM-MSCs	(20)
	LD formation	miR-1908-5p	PLIN4	—	—	—	(31)
	Obesity	miR-1908-5p	—	PPARγ, C/EBPα	—	promote hMADS cell growth and proliferation, and inhibit differentiation of hMADS cells to form mature adipocytes	(33)
	LDL-C in circulation	miR-1908-5p	TGF-β1	BMP1	—	reduce ratio of LDLR cut mature to full-length LDLR and promote LDL-C uptake by liver cells (HuH-7)	(11)
	AMI	miR-1908-5p	TGF-β1	smad2/3	improve cardiac function and reduce myocardial fibrosis after infarction	—	(1)
	RA	miR-1908-5p	STAT3	—	reduce synovial inflammation and cartilage erosion	inhibit differentiation of Th17, decrease the proportion of Th17 cells and increase the proportion of Treg cells in CD4+ T cell population	(19)
	Renal fibrosis	miR-1908-5p	TGF-β1	smad2/3	alleviate progression of renal fibrosis	inhibit fibrosis of renal interstitial cell (HEK293A)	(7)
	Scar formation post-burn	miR-1908-5p	Ski	TGF-β	accelerate scar formation	promote fibrosis and scar formation	(32)
	BD	miR-1908-5p	GRM4	—	—	—	(39)
		miR-1908-5p	KLC2	—	—	—	(40)
	AD	miR-1908-5p	ApoE	Aβ	—	impair capacity of macrophage (THP-1) and astrocytes (U87) to clear Aβ	(15)

NSCLC, non-small cell lung cancer; EOC, epithelial ovarian cancer; PCa, prostate cancer; SSCs, spermatogonial stem cells; LD, lipid droplet; LDL-C, low-density lipoprotein cholesterol; AMI, acute myocardial infarction; RA, rheumatoid arthritis; BD, bipolar disorder; AD, Alzheimer’s disease; BM-MSCs, bone marrow-derived mesenchymal stem cells; LDLR, low-density lipoprotein receptor.

### Regulation of miR-1908 on the Proliferation of Cancer Cells

In different cancers, miR-1908 has different regulatory effects on cell proliferation. miR-1908 promotes cell proliferation in glioma, hMADS cells, breast cancer, and human SSCs while inhibiting cell proliferation in NSCLC and chordoma. In addition, there are still inconsistent results regarding the effect of miR-1908 on cell proliferation in osteosarcoma.

**Figure 2:**
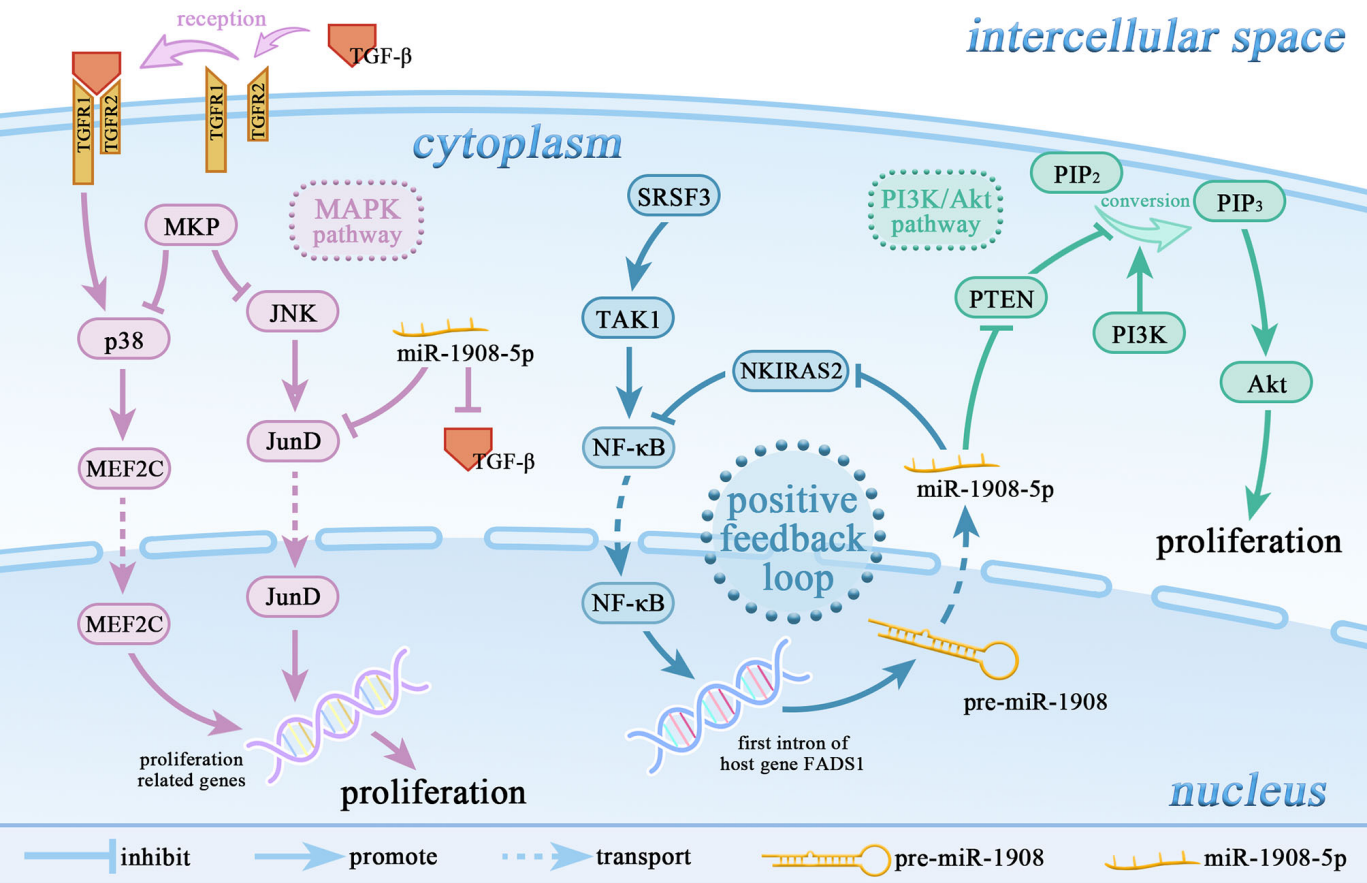
miR-1908-5p is involved in the regulation of multiple signaling pathways. miR-1908-5p plays an important role in the MAPK signaling pathway and PI3k/Akt signaling pathway. The positive feedback loop of NF-κB/miR-1908-5p/NKIRAS2/NF-κB enhances the promoting effect of the SRSF3/TAK1/NF-κB axis on the expression of miR-1908-5p.

miR-1908 can promote cell proliferation by targeting 11 genes, including PTEN, SPRY4, ID4, LTBP4, GPM6B, RGMA, EFCAB1, ALX1, OSR1, PPAPA, and KLF2. As shown in **Figure 2**, miR-1908-5p can target the tumor suppressor gene PTEN, activate the PI3K/AKT signaling pathway, and then promote the proliferation and angiogenesis of 12 types of glioma cells including A127 (8). At the same time, it can also target SPRY4, promote the expression of the proto-oncogene RAF1, and further promote the development of glioma (13). In hMADS cells, miR-1908-5p can induce cells in the G1 phase to enter the S phase, thereby promoting the proliferation and growth of hMADS cells (33). miR-1908-3p promotes the proliferation of breast cancer MCF-7 cells by inhibiting the expression levels of 8 target genes such as ID4 (37). miR-1908-3p promotes the long-term non-tumorigenic proliferation of human SSCs by targeting KLF2, reflecting that miR-1908-3p is a regulatory factor that maintains the stemness of human SSCs (30).

miR-1908 can inhibit cell proliferation by targeting genes such as AKT1S1, PP5, TGF-β1, and JunD. In most cancers, high expression of miR-1908-5p usually promotes cancer cell proliferation. However, the expression level of miR-1908-5p is decreased in non-small cell lung cancer (NSCLC). In a mildly hypoxic environment, miR-1908-5p targets AKT1S1 to up-regulate the expression of marker genes in the RP/p53/p21 signaling axis and inhibit the proliferation of NSCLC cells (including SK-MES-1, A549, and NCI-H460) (22). Experimental results in athymic mice and SPC-A1 cell lines show that miR-1908-5p targets PP5 and inhibits the proliferation of NSCLC cells (23). In addition, as shown in **Figure 2**, in chordoma, miR-1908-5p can target TGF-β1 and JunD, inhibit the MAPK signaling pathway, and thereby hinder the proliferation of cancer cells (6).

In osteosarcoma, the effect of miR-1908 on cancer cell proliferation still has inconsistent results. miR-1908-5p can activate the PI3K/AKT signaling pathway by inhibiting PTEN, thereby promoting the proliferation of osteosarcoma cells (143B, MG63, SAOS2, and U2OS) (9). But on the contrary, another study found that miR-1908-5p can target ROCK1, thereby inhibiting the proliferation of osteosarcoma cells (HOS, MG293, G63, SAOS2, and U2OS) (38). In the future, more rigorous research is needed to clarify the reasons for the inconsistent results of miR-1908 in osteosarcoma.

### The Role of miR-1908 in Cell Differentiation

Cell differentiation can produce cells with different structures and different functions (41). miR-1908-5p can target and inhibit the expression of EXO1, PLIN4, and STAT3, and indirectly hinder the transcription of PPARγ and C/EBPα genes, thereby promoting the differentiation of bone marrow mesenchymal stem cells, adipocytes, CD4^+^ T cells, and hMADS cells. In patients with type 2 diabetes (T2DM), miR-1908-5p can target EXO1, thereby inhibiting the proliferation and osteogenic differentiation of bone marrow mesenchymal stem cells, thereby increasing the risk of fractures (20). Lipid droplets (LD) are an important subcellular organelle, which has the function of storing neutral lipids and replenishing energy by releasing fatty acids. During the formation of LD, miR-1908-5p targets PLIN4, which is involved in adipocyte differentiation (31). In RA, miR-1908-5p is down-regulated by competitive inhibition of lncRNA HOTTIP. Overexpression of miR-1908-5p can target STAT3 and inhibit Th17 cell differentiation, leading to a decrease in the proportion of Th17 cells and an increase in the proportion of Treg cells in the CD4^+^ T cell population, and then reduce inflammation (19). In another study, miR-1908-5p can promote the growth of hMADS cells, and inhibit the differentiation of adipocytes by blocking the transcription of adipogenesis-determining genes PPARγ and C/EBPa (33).

### The Dual Role of miR-1908 in Cell Apoptosis

Apoptosis is a programmed death triggered by multiple biological factors and mediated by multiple signaling pathways (42). Phagocytosis and clearance of apoptotic cells by macrophages are killed by caspase family proteins, avoiding the release of intracellular components and inflammatory factors (43). Apoptosis defect is closely related to the occurrence and development of cancer and the resistance of cancer cells to chemotherapeutic drugs (44, 45). Apoptosis plays a very important role in a variety of tumors (45).

miR-1908-5p can simultaneously target the anti-apoptotic protein PP5, and inhibit the pro-apoptotic protein Bax, thereby playing a dual role in regulating apoptosis. In EOC, miR-1908-5p can significantly increase the apoptosis of two cell lines A2780 and SK-OV-3 (28). The anti-apoptotic protein Bcl-2 strictly controls the entry of cytochrome c from the mitochondrial intermembrane space into the cytoplasm by regulating the expression level of the effector protein Bax (46). Cytochrome c released into the cytoplasm can promote the generation and activation of apoptotic bodies, and further facilitate the maturation of caspase family proteins, and promotes apoptosis (47). In glioma, miR-1908-5p can promote the expression of anti-apoptotic protein Bcl-2 and down-regulate the expression of pro-apoptotic protein Bax, thereby inhibiting the activation of apoptotic body pathway and the level of caspase family proteins, enhancing the anti-apoptotic capacity of U251 cells (13). In NSCLC, miR-1908-5p promotes SPC-A1 cell apoptosis by targeting the anti-apoptotic protein PP5 (23).

### The Role of miR-1908 in Cancer Cell Invasion and Metastasis

Tumor metastasis is an invasion-metastasis cascade process. Its main steps are divided into the local invasion and breakthrough of the basement membrane by cancer cells, intravasation and invasion of blood vessels and lymphatic vessels, and then the process of proliferation, proliferation, and colonization, and finally the formation of secondary tumors (48, 49). Tumor metastasis is an important reason for the high recurrence and mortality of cancer (50). miR-1908 regulates the invasion and metastasis of various cancer cells by regulating the expression of target genes.

**Figure 3:**
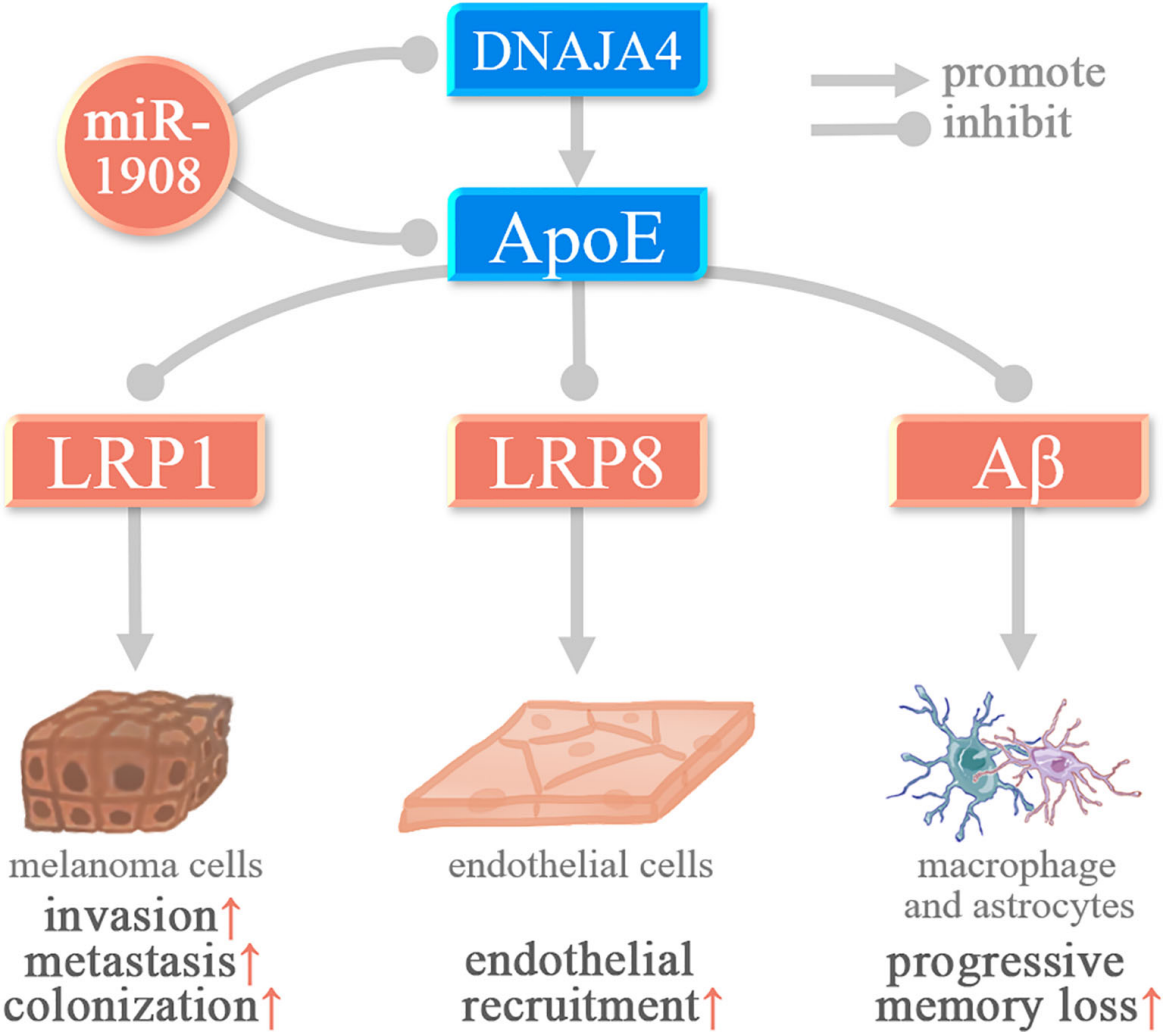
miR-1908-5p plays a role in melanoma and Alzheimer’s disease by targeting ApoE.

Among the target genes of miR-1908-5p, PTEN, DNAJA4, ApoE, and HDAC10 were associated with cancer cell invasion and metastasis. In glioma and osteosarcoma, miR-1908-5p activates the PI3K/AKT signaling pathway by targeting the tumor suppressor gene PTEN and promotes cancer cell proliferation, invasion, spheroid formation, and angiogenesis (8, 9). In glioma, activation of AKT/FOXO3a and AKT/mTOR signaling pathways mediates the promotion of miR-1908-5p on the malignant phenotype of glioma cells U87. As shown in **Figure 3**, in melanoma, miR-1908-5p can target DNAJA4 and ApoE to promote the invasion and metastasis of MeWo-LM2 cells. Among them, DNAJA4 can positively regulate ApoE, and extracellular ApoE can target the LRP1 receptor of melanoma cells and the LRP8 receptor of endothelial cells, thereby inhibiting cancer cell invasion, metastasis, colonization, and endothelial recruitment (35). In cervical cancer, miR-1908-5p promotes metastasis and colony formation of Ca-Ski, SiHa, and C-4I cancer cells by targeting HDAC10 (26). In breast cancer, miR-1908-3p promotes the invasion and metastasis of breast cancer MCF-7 cells by targeting eight genes including ID4, thereby promoting the progression of breast cancer (37).

### miR-1908 Regulates the Secretion of Extracellular Vesicles

Extracellular vesicles (EV) are micro-membrane vesicles actively released by cells, and they are an important carrier for cell-to-cell communication. miR-1908 can regulate cell secretion of EV. In prostate cancer (PCa) tissues, the expression level of spermidine synthase (SRM) is elevated. SRM can promote EV secretion in 22Rv1 cells, thereby regulating the cancer microenvironment and promoting cancer progression. miR-1908-5p can target SRM to regulate EV secretion and inhibit the pathogenesis of PCa (29). Exosomes are a type of EV, a new type of mediator for tumorigenesis and immune escape. In nasopharyngeal carcinoma (NPC), miR-1908-5p is overexpressed in TW03 (EBV-) cell exosomes, which can down-regulate the MARK1 signaling pathway, thereby promoting the proliferation and differentiation of NPC cells (21).

## The Molecular Mechanism of miR-1908 in Human Diseases

**Figure 4:**
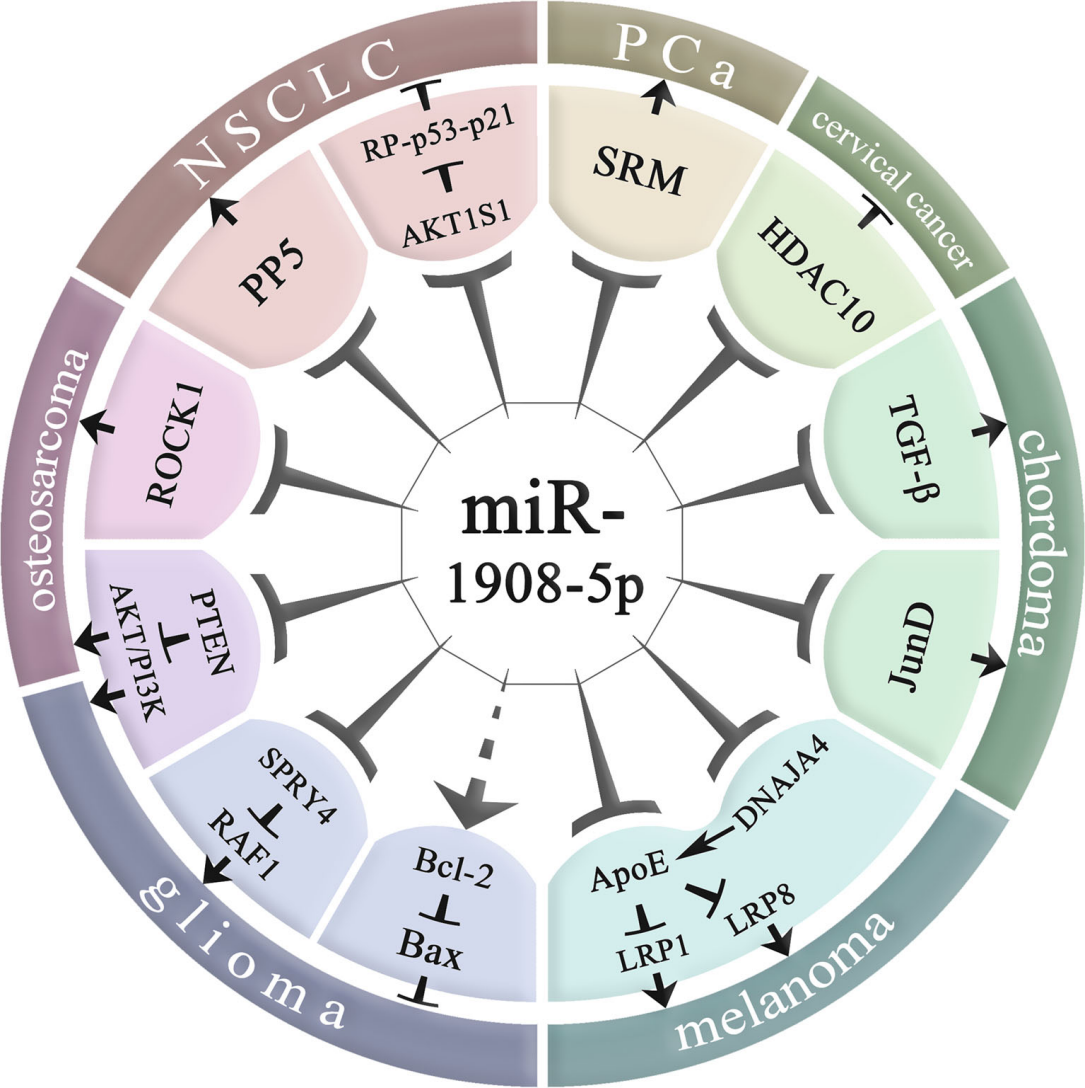
Molecular regulatory mechanisms of miR-1908-5p in cancer. NSCLC, non-small cell lung cancer; PCa, prostate cancer; miR-1908-5p plays an important role in various cancers by regulating downstream gene expression.

As shown in **Table 2** and **Figure 4**, miR-1908 not only plays an important role in the pathogenesis of cancer but is also associated with the risk of four non-cancer diseases, including organ and tissue fibrosis (1, 7, 32), bipolar disorder (BD) (39, 40), Alzheimer’s disease (AD) (15), RA (19). At the same time, miR-1908 is also involved in the regulation of lipid metabolites (51) and ABH antigen (52).

### The Molecular Mechanism of miR-1908 in Cancer

MiR-1908 plays a role in many aspects of cancer biology, including proliferation, apoptosis, invasion and metastasis, and angiogenesis, and plays an important role in the occurrence and development of cancer. Abnormally high expression of miR-1908 can promote the development of glioma, cervical cancer, PCa, chordoma, melanoma, and breast cancer, but it inhibits the risk of NSCLC. In addition, the role of miR-1908 is inconsistent in osteosarcoma. The relationship between miR-1908 and cancer susceptibility is race-specific (53).

MiR-1908-5p promotes the proliferation, differentiation, invasion, and metastasis of glioma cancer cells by targeting PTEN, and SPRY4, and inhibits the apoptosis of glioma cancer cells (8, 13). miR-1908-5p targets HDAC10 to promote the growth, invasion, and metastasis of cervical cancer cells (Ca-Ski, SiHa, and C-4I) (26). miR-1908-5p can target SRM to regulate extracellular vesicle (EV) secretion (29), thereby promoting PCa cancer progression (54). miR-1908-5p targets TGF-β1 and JunD in the MAPK pathway to promote the proliferation of chordoma tissue (6). miR-1908-5p targets APOE and DNAJA4 and promotes the invasion and metastasis of melanoma cells (MeWo-LM2) (35). miR-1908-3p targets 8 genes including ID4 and promotes the proliferation, invasion, and metastasis of breast cancer cells (MCF-7) (37).

In contrast to the above, miR-1908-5p targets AKT1S1 and PP5 in NSCLC, inhibits cancer cell proliferation and promotes cancer cell apoptosis (22, 23). In addition, miR-1908-5p may have two-way functions in osteosarcoma. On the one hand, miR-1908-5p targets PTEN to promote the proliferation, invasion, and metastasis of osteosarcoma cells such as 143B, U2OS, MG63, and SAOS2 (9); on the other hand, miR-1908-5p can also target ROCK1 and inhibit the proliferation of osteosarcoma cells such as HOS, MG63, G293, SAOS2, and U2OS (38). It is worth noting that the mode of action of miR-1908 in osteosarcoma is still not fully understood. miR-1908 can not only inhibit the development of osteosarcoma by targeting ROCK1, but also activate the PI3K/AKT signaling pathway to promote the development of osteosarcoma.

The role of miR-1908 in different cancers may be due to the differential expression of miR-1908-related genes in different tissues. In different cancers, the upstream factors of miR-1908 are differentially expressed, resulting in differences in the downstream regulation patterns of miR-1908. In the future, more in-depth studies are still needed to understand the molecular mechanism by which miR-1908 plays a role in cancer, to provide a theoretical basis for miR-1908-targeted therapy.

### The Role of miR-1908 in Tissue and Organ Fibrosis

**Figure 5:**
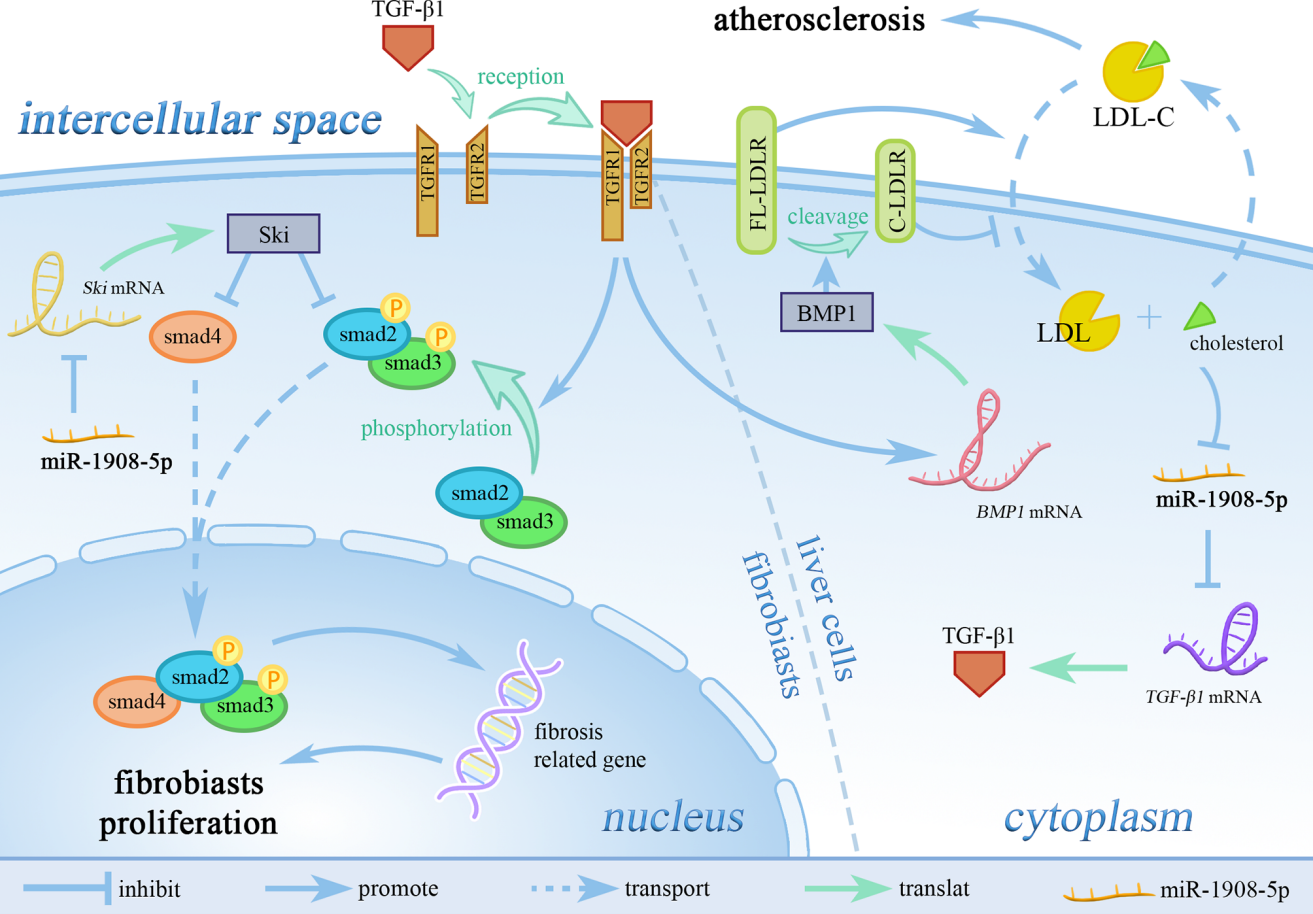
miR-1908-5p regulates LDL-C metabolism in blood through the TGF-β1 signaling pathway. TGFR1, TGF receptor 1; TGFR2, TGF receptor 2; LDL-C, low-density lipoprotein cholesterol; FL-LDLR, full-length LDL receptor; C-LDLR, cleaved LDL receptor. miR-1908-5p plays an pivotal role in the process of organ and tissue fibrosis and atherosclerosis by targeting TGF-β1.

Fibrosis is a pathological process that occurs in organ tissues with excessive accumulation of extracellular matrix, and the gradual decrease of substantive functional cells, which eventually leads to organ dysfunction (7). As shown in **Figure 5**, in the myocardial border zone and renal interstitial tissue of patients with renal fibrosis after myocardial infarction, miR-1908-5p can inhibit the TGF-β1-smad2/3 signaling pathway by targeting TGF-β1 to reduce the key phosphorylation of intracellular signaling protein smad2/3, thereby alleviating organ fibrosis and improving organ function (1). At the same time, in renal interstitial cells overexpressing miR-1908-5p, the expression level of the important extracellular matrix hydrolase MMP-2 and the cell proliferation rate were significantly diminished (7). After skin burns, the expression of miR-1908-5p is increased in wound tissues, and by targeting to inhibit Ski, it promotes the formation of scars (32).

### The Molecular Mechanism of miR-1908 in Alzheimer’s Disease

Alzheimer’s disease (AD) is a common disease that affects memory and thinking skills (55). Aβ gradually precipitates and accumulates in cells, causing neurotoxicity, which in turn leads to a series of AD symptoms such as memory loss and progressive neurologic deterioration (56). In AD, miR-1908-5p can target ApoE, thereby inhibiting ApoE-mediated β-amyloid (Aβ) clearance (15).

### miR-1908 Regulates the Level of Lipid Metabolites

High expression levels of miR-1908-5p are closely related to low levels of low-density lipoprotein (LDL), total cholesterol, fasting glucose, and glycosylated hemoglobin (51). The high expression of miR-1908-5p is significantly related to the decrease of LDL-C levels in the population. As shown in **Figure 5**, miR-1908-5p does not affect the expression of low-density lipoprotein receptor (LDLR) but can reduce the expression of bone morphogenetic protein 1 (BMP1) by targeting TGF-β1. Down-regulation of BMP1 expression can increase the stability of LDLR, which in turn promotes the absorption of LDL-C by liver cells, thereby reducing circulating low-density lipoprotein-cholesterol (LDL-C) levels (11), and ultimately reducing the risk of cardiovascular disease (57).

**Figure 6:**
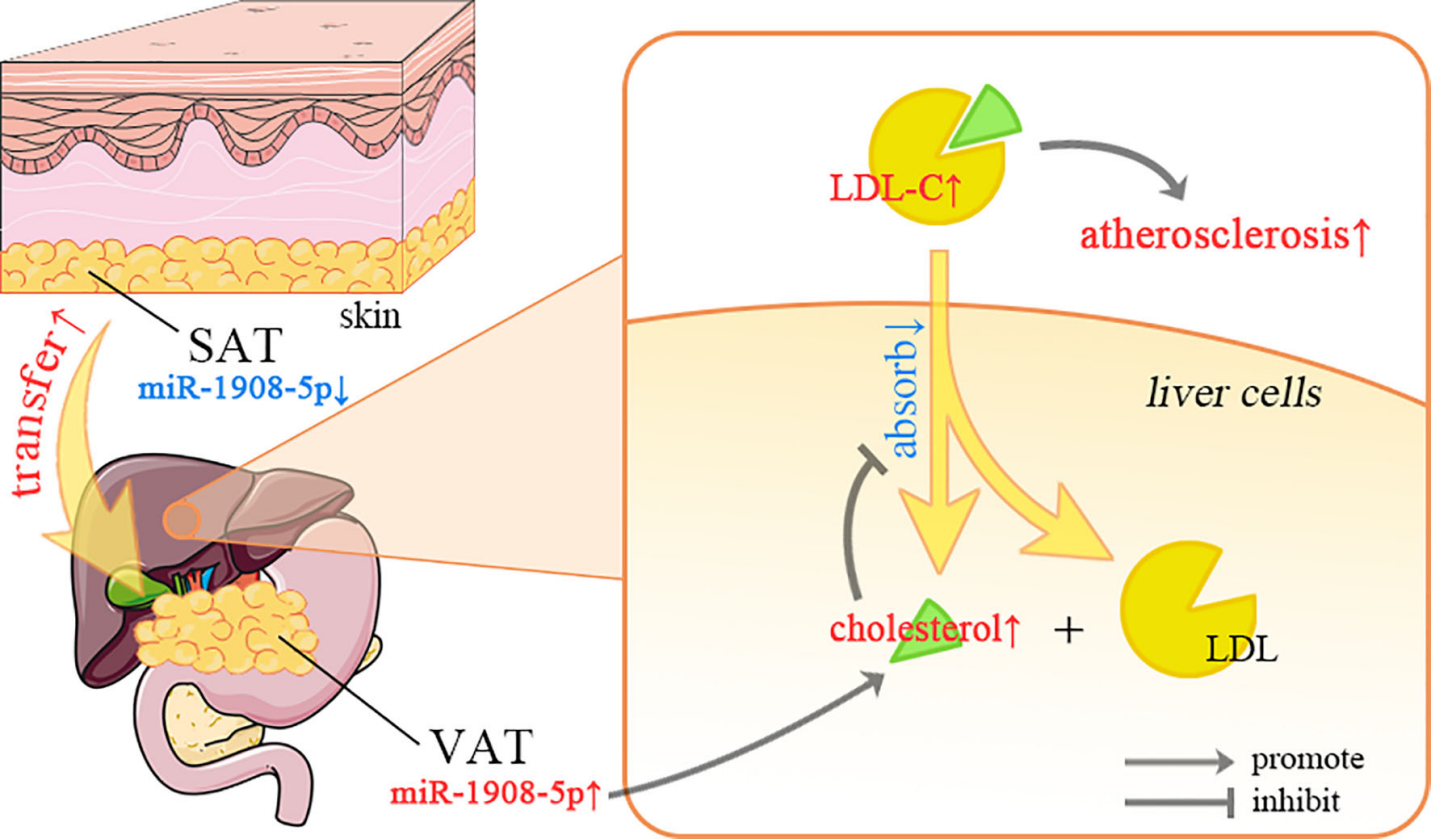
The regulation of miR-1908-5p in lipid metabolism. SAT, subcutaneous adipose tissue; VAT, visceral adipose tissue; LDL-C, low-density lipoprotein cholesterol; The differential expression of miR-1908-5p in different organs may be related to the distribution of adipose tissue, and its abnormal expression may be related to fatty liver and atherosclerosis.

In the initial stage of obesity, fat is mainly deposited under the skin, and then gradually transferred to the internal organs (58). The correlation between visceral adipose tissue (VAT) and cardiovascular disease is stronger than that of subcutaneous adipose tissue (SAT) (59). As shown in **Figure 6**, the different miR-1908-5p expression patterns between VAT and SAT may facilitate this process. In the serum of most IS patients, the expression level of miR-1908-5p is down-regulated (18). This may be due to the overload of cholesterol in liver cells inhibiting the expression of miR-1908-5p. The low expression level of miR-1908-5p further inhibits the recovery of LDL-C from the blood by hepatocytes and promotes the occurrence of atherosclerosis (11).

## miR-1908 Is Involved in Multiple Cancer-Related Signaling Pathways

The current study shows that miR-1908 is involved in the regulation of the MAPK signaling pathway (6), TGF-β signaling pathway (1, 7), PI3K/AKT signaling pathway (8, 9), and AMPK signaling pathway (10).

### The MAPK Signaling Pathway

The mitogen-activated protein kinase (MAPK) signaling pathway regulates cell growth, proliferation, and apoptosis through Jun N-terminal kinase (JNK) and p38 (60–62). In chordoma, miR-1908-5p targets TGF-β and JunD, thereby inhibiting the JNK and p38 MAPK signaling. Downregulation of miR-1908-5p can lead to aberrant activation of the MAPK signaling pathway, thereby promoting the occurrence of cancer (6).

### The TGF-β Signaling Pathway

The transforming growth factor β (TGF-β) signaling pathway can regulate cell proliferation, differentiation, migration, apoptosis, and EMT (63, 64). SMAD proteins play a role in signal transduction and transcriptional regulation in the TGF-β signaling pathway (65). Smad2/3 proteins are phosphorylated upon TGF-β signaling, bind to Smad4, and enter the nucleus as transcription factors to induce cell proliferation, migration, and EMT (65, 66). Studies have shown that the targeted inhibition of TGF-β by miR-1908-5p can reduce the phosphorylation level of downstream smad2/3 proteins, thereby inhibiting the process of cardiac/kidney fibrosis (1, 7), or reducing the level of BMP1 and promoting blood LDL-C levels, which are then taken up by hepatocytes (11).

The TGF-β signaling pathway plays a bidirectional role in cancer. TGF-β can not only inhibit tumor progression by promoting apoptosis, but also promote cancer cells to initiate immune evasion, invasion, and metastasis (67). The inhibitory effect of miR-1908 on the TGF-β signaling pathway may also play a role in cancer.

### The PI3K/AKT Signaling Pathway

The phosphoinositide 3-kinase (PI3K)/protein kinase B (Akt) signaling pathway is primarily responsible for cellular responses to changes in the extracellular environment and plays a regulatory role in proliferation, differentiation, apoptosis, and metabolic activities (68). The tumor suppressor gene PTEN is a specific phosphatase of PIP3, which can dephosphorylate PIP3 to generate 4,5-bisphosphate (PIP2), resulting in the inability of Akt to be recruited and activated on the plasma membrane, thereby inhibiting the PI3K/Akt signaling pathway (69). In glioma and osteosarcoma, miR-1908-5p can target and inhibit the expression level of PTEN and promote the activation of the PI3K/Akt signaling pathway, thereby promoting cancer cell proliferation and angiogenesis (8, 9).

### The AMPK Signaling Pathway

AMP-activated protein kinase (AMPK) is a kinase related to cellular metabolism. Activation of the AMPK signaling pathway can promote catabolism and inhibit anabolism, reducing the intracellular AMP/ATP ratio (70). Dysregulation of energy metabolism is an important reason for inducing cancer, and abnormal activation of the AMPK signaling pathway is considered to be closely related to the occurrence and development of cancer (71). LKB1 is an important upstream kinase of the AMPK signaling pathway that controls cell metabolism and growth in tumor suppression (72). In liver cancer cell HuH-7, miR-1908-5p could downregulate the expression of LKB1 and further inhibit the activity of AMPK (10). This may be related to the occurrence of obesity, but the mechanism of miR-1908-5p in the process of hepatocarcinogenesis is still not fully understood.

## The Regulation Mechanisms of miR-1908 Expression

As shown in **Table 3**, the expression of miR-1908 is also regulated by 11 factors, including lncRNA HOTTIP, adipokines (TNF-α, leptin, and resistin), NF-κB, FFA, cholesterol, SCD1, and immune-related transcription factors (STAT1, RB1, and IRF1).

**Table 3:** Upstream factors of miR-1908-5p.

Disease	Upstream factors	miR-1908-5p	Reference
Osteosarcoma	SRSF3/TAK1/NF-κB	Up-regulation	(73)
RA	lncRNA HOTTIP	Down-regulation	(19)
CD and/or RA	STAT1, RB1, IRF1	Down-regulation	(74)
Low-energy fracture	SCD1	Down-regulation	(20)
Atherosclerosis	cholesterol	Down-regulation	(11)
Obesity	TNF-α/NF-κB	Up-regulation	(34)
	leptin, resistin, FFA	Down-regulation	
	NF-κB	Down-regulation	(4)

CD, Crohn’s disease; RA, rheumatoid arthritis.

Competing for endogenous RNAs (ceRNAs) is a major molecular mechanism for long noncoding RNAs (lncRNAs). Among them, lncRNAs up-regulate the expression levels of miRNA target genes by competitively inhibiting the activity of miRNAs (75, 76). LncRNA HOTTIP can sponge miR-1908, thereby increasing the expression level of STAT3. The HOTTIP/miR-1908-5p/STAT3 axis can promote Th17 cell proliferation and activate Th17 cell differentiation. Subsequently, the imbalance of Th cells can aggravate synovial inflammation and cartilage erosion, thereby causing RA (19).

In addition, TNF-α can significantly increase the expression of miR-1908-5p in human adipocytes. In contrast, leptin and resistin can significantly reduce the expression of miR-1908-5p after treating human adipocytes (34).

There are two potential binding sites for NF-κB at the -1229bp and -383bp positions of the miR-1908 promoter (4). In human adipocytes, TNF-α activates NF-κB, thereby increasing the expression level of miR-1908-5p (34). As shown in **Figure 2**, in osteosarcoma U2OS cells, the proto-oncogene SRSF3 up-regulates the expression of TAK1, further activates NF-κB, and up-regulates the expression of miR-1908-5p, which ultimately stimulates the proliferation, invasion, and metastasis of cancer cells. Meanwhile, miR-1908-5p can hinder the expression of NKIRAS2 to activate the transcriptional activity of NF-κB. The positive feedback regulation of NF-κB/miR-1908-5p/NKIRAS2/NF-κB can greatly enhance the tumorigenicity of SRSF3 (73).

During the maturation of HPA-V, the expression of miR-1908-5p is not related to that of its host gene FADS1. After NF-κB activator TNF-α treats HPA-V, the expression level of miR-1908-5p decreases, while after NF-κB inhibitor JSH-23 treats HPA-V, the expression level of miR-1908-5p increases. The above results indicate that NF-κB can inhibit the expression of miR-1908-5p in HPA-V (4).

In addition, in human adipocytes, free fatty acids (FFA) can continuously reduce the expression level of miR-1908-5p (34). In liver cells, overloaded cholesterol can also inhibit the expression of miR-1908-5p (**Figure 5**) (11).

In patients with T2DM, stearoyl-CoA desaturase-1 (SCD1) can inhibit the expression of miR-1908-5p and up-regulate the expression of EXO1, thereby promoting the osteogenic differentiation of bone marrow mesenchymal stem cells and ultimately inhibiting the occurrence of fractures in T2D patients (20).

In autoimmune diseases such as Crohn’s disease (CD) and RA, transcription factors such as STAT1, RB1, and IRF1 are closely related to immunity and can hinder the expression of miR-1908-5p (74).

## The Potential Role of miR-1908 in the Treatment of Human Diseases

Many studies have shown that miR-1908 is closely related to the occurrence and development of human diseases. At the same time, miR-1908 can be potentially used as a biomarker to predict the therapeutic outcomes of patients.

### The Correlation Between miR-1908 and Drug Efficacy

**Figure 7:**
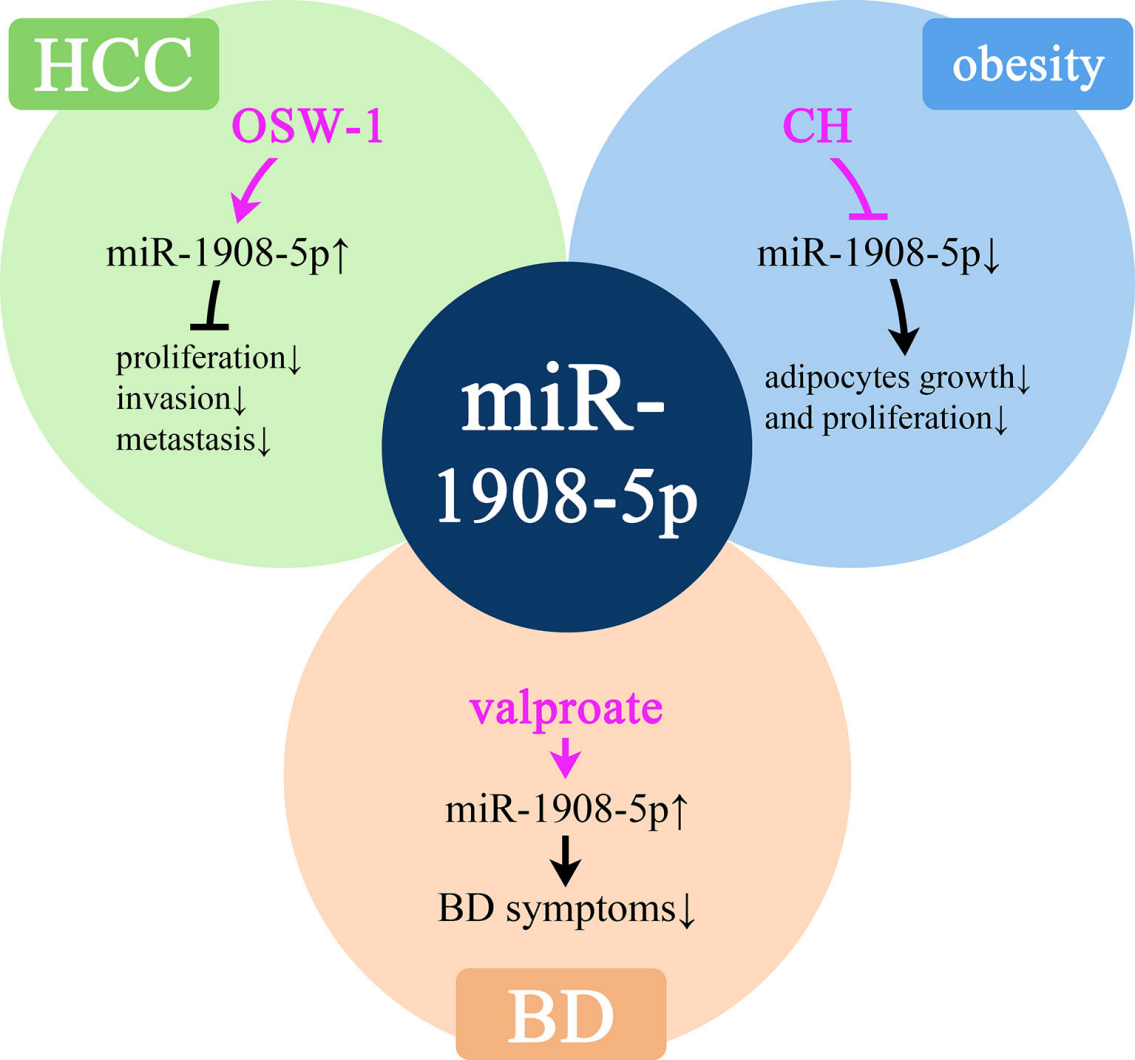
Molecular mechanisms of potential drugs that can modulate miR-1908-5p. HCC, hepatocellular carcinoma; BD, bipolar disorder.

In HCC, obesity, and BD, miR-1908 can respectively inhibit the activity of cancer cells, promote fat conversion, and cause emotional excitement. As shown in **Figure 7**, a variety of drugs have affected the expression level of miR-1908 in these diseases.

OSW-1 is an anticancer drug of cholestane saponins. A study found that after OSW-1 treatment of liver cancer Hep3B cells, the expression of miR-1908-5p increased by 5 times, thereby inhibiting the proliferation, invasion, and metastasis of cancer cells, thereby exerting anti-cancer effects (77). miR-1908-5p plays an important role in obesity-related complications. In human adipocytes, growth hormone (GH) can significantly inhibit the expression of miR-1908-5p in a short time and then promote lipolysis (34). In BD patients, the expression level of miR-1908-5p is low. Sodium valproate is a commonly used drug for the treatment of BD. Treatment with sodium valproate can significantly increase the expression of miR-1908-5p, thereby reducing the symptoms of BD (39). At the same time, in the plasma of OC patients, the high expression of miR-1908-5p is significantly associated with platinum resistance (78).

### The Prognostic Value of miR-1908 in Cancer

The abnormal expression of miR-1908 is also closely related to the patient’s pathological conditions. As shown in **Table 4**, miR-1908-5p can be used as a biomarker for the diagnosis and prognosis of various diseases.

**Table 4:** Prognostic value of miR-1908 in different diseases.

Disease	Type of miRNA	Sample	Prognosis of miR-1908 over-expression	Prognosis type	Reference
Breast cancer	miR-1908-3p	60 patients (GSE33447)	poor	OS	(37)
Cervical cancer	miR-1908-5p	36 patients (GSE63514)	poor	OS	(26)
Glioma	miR-1908-5p	206 patients from TCGA database	poor	OS, DFS	(13)
	miR-1908-5p	24 Grade III and 50 Grade IV patients (GSE4412)	good	OS	(14)
	miR-1908-5p	47 patients	poor	DFS	(8)
NSCLC	miR-1908-5p	76 patients	good	5YSR	(23)
OC	miR-1908-5p	491 patients from TCGA database	good	OS, DFS	(79)
HGSOC	miR-1908-5p	175 patients (GSE106817)	poor	OS, PFS	(28)
Osteosarcoma	miR-1908-5p	212 patients	poor	OS, DFS	(12)
	miR-1908-5p	46 patients	poor	OS	(9)

NSCLC, non-small cell lung cancer; OC, ovarian cancer; HGSOC, high-grade serous ovarian carcinoma; GEO, the Gene Expression Omnibus; TCGA, the Cancer Genome Atlas; NCBI, National Center for Biotechnology Information; OS, overall survival; DFS, disease-free survival; 5YSR, 5-year survival rate; PFS, progression-free survival.

In cervical cancer, HGSOC, osteosarcoma, and breast cancer, patients, high expression of miR-1908-5p is significantly associated with lower overall survival (OS) (9, 26, 28, 37). In HGSOC patients, high serum miR-1908-5p expression is significantly associated with poor progression-free survival (PFS) (28). In patients with osteosarcoma, high expression of miR-1908-5p points to poor disease-free survival (DFS). In addition, the high expression of miR-1908-5p is also significantly associated with poor chemotherapy efficacy and a higher recurrence rate in patients with osteosarcoma (12).

In contrast to the above, in NSCLC, patients with low miR-1908-5p expression have a lower five-year survival rate (23). In PCa patients, higher miR-1908-5p expression may indicate a better prognosis for patients (29). In OC, patients with low miR-1908-5p expression have lower OS and DFS (79).

In addition, there are inconsistencies in the correlation between the expression level of miR-1908-5p and the prognosis of brain tumors. Among the 47 patients with glioblastoma, patients with high miR-1908-5p expression had shorter DFS (8). Among 206 patients with TCGA glioma, patients with high expression of miR-1908-5p had shorter OS and DFS (13). In contrast, among 24 patients with grade III glioma and 50 patients with grade IV glioma from the GEO database (GSE4412), patients with low expression of miR-1908-5p had a shorter OS (14).

MiR-1908 has different prognostic roles in various cancers. More experimental evidence is needed in the future to further demonstrate the role of miR-1908 in cancer therapy, diagnosis, and prognosis.

## Conclusions

This work shows that miR-1908 is aberrantly expressed in many diseases, especially cancer. miR-1908 can downregulate the expression of at least 27 target genes by binding to the 3-’UTR of its mRNA. miR-1908 is widely involved in a variety of cellular behaviors, including cell proliferation, cell differentiation, apoptosis, cancer cell invasion and metastasis, and extracellular vesicle secretion. miR-1908 is regulated by multiple upstream factors, including ceRNAs, transcription factors, hormones, energy metabolites, and drugs. miR-1908 is an important intermediate signal transduction molecule, suggesting that miR-1908 may be a potential drug therapy target for cancer. The systematic overview of miR-1908 in this work provides clues and directions for future research on miR-1908.

## Author Contributions

JS, YW, WR, and FZ collected and analyzed the literature, drafted the figures and wrote the paper; SD and FZ conceived and gave the final approval of the submitted version. All authors have read and agreed to the published version of the manuscript.

## Funding

This study was supported by the Medical and Health Science and Technology Program of Zhejiang Province (No. 2020RC088).

## Conflict of Interest

The authors declare that the research was conducted in the absence of any commercial or financial relationships that could be construed as a potential conflict of interest.

## Publisher’s Note

All claims expressed in this article are solely those of the authors and do not necessarily represent those of their affiliated organizations, or those of the publisher, the editors and the reviewers. Any product that may be evaluated in this article, or claim that may be made by its manufacturer, is not guaranteed or endorsed by the publisher.
